# Cable deployment system for unmanned ground vehicle (UGV) mobile microgrids

**DOI:** 10.1016/j.ohx.2021.e00205

**Published:** 2021-05-23

**Authors:** John E. Naglak, Caleb Kase, Max McGinty, Casey D. Majhor, Carl S. Greene, Jeremy P. Bos, Wayne W. Weaver

**Affiliations:** a Department of Mechanical Engineering-Engineering Mechanics, Michigan Technological University, Houghton, MI, USA; b Department of Electrical and Computer Engineering, Michigan Technological University, Houghton, MI, USA

**Keywords:** Tether, Electrical connection, Cable management

## Abstract

An ad hoc autonomous mobile microgrid system requires electrical connections to be formed between physically separated resources. This work proposes the use of unmanned ground vehicles (UGVs) as the means to deploy the electrical cable that creates these connections. This operation requires careful control of the cabling at variable speeds to avoid entanglement with the deploying UGV or obstacles in complex outdoor environments. Searching for a product that could supply the needed control and flexibility revealed a lack of compact and low-cost options. Existing options are very heavy (>100 lbs) and do not supply precision in their deployment. There is no commercial off-the-shelf option available for small-scale cable deployment operations with size and weight constraints. To fulfill the application requirements and to combat this deficiency, a custom design and build of an “Adjustable Cable Management Mechanism” (ACMM) was required. This ACMM provides a low cost, compact platform for powered and controlled deployment and retraction of different-sized cable under moderate loads, utilizing Commercial Off-The-Shelf components (COTS). Employing this design has enabled a variety of tasks that require distribution of electrical or data cables to be accomplished for small-scale projects. The goal of this paper is to give detailed design specifications of the ACMM and instructions on how to recreate it and calibrate it to be useful for tethering robots in various applications such as steep terrain, internet connection through tight spaces, or electrical connection between nodes for complex microgrids.

## Graphical abstract

**Figure:**
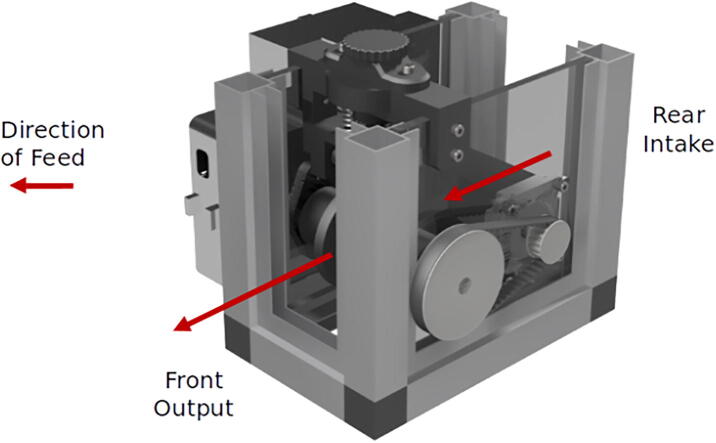

Specifications table:

**Table:** 

**Hardware name**	Adjustable Cable Management Mechanism
**Subject area**	Engineering and Material Science
**Hardware type**	Mechanical engineering and materials science
**Open source license**	GNU GPL and CERN OHL
**Cost of hardware**	USD434:23
**Source file repository**	https://doi.org/10.17605/OSF.IO/8WKJT

## 1 Hardware in context

**Fig. 1:**
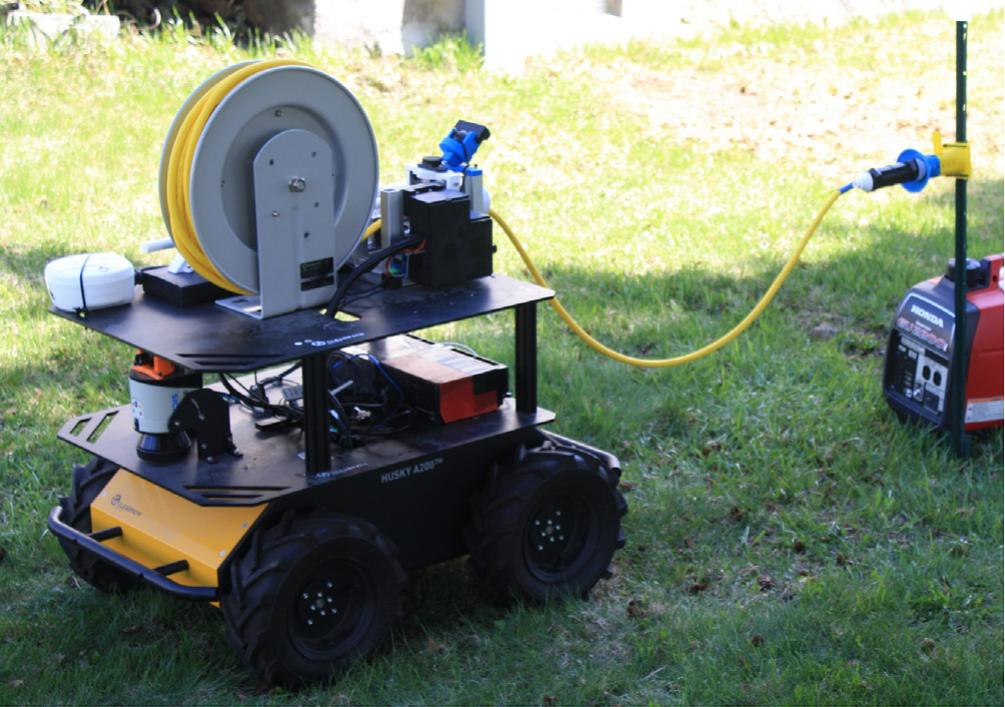
An Autonomous Mobile Microgrid UGV deploys a power cable to connect a genset power source to an infrastructure load.

A microgrid is a system of interconnected, distributed energy resources, which creates a localized power infrastructure. Microgrid connections depend on the connection of two or more nodes with wired electrical connections. These nodes include energy sources and critical loads. An autonomous mobile microgrid employs many of the same resources as a traditional microgrid, but has the added benefits of mobility, self-organization, and re-configuration by autonomous agents. Unmanned Ground Vehicles (UGVs), equipped with energy generation, power control, cable coupling, and routing mechanisms, can establish this power infrastructure and transmission [1]. These autonomous mobile microgrids have many beneficial applications such as military forward operating bases, planetary operating bases, and disaster recovery [2], [3], [4], relieving humans from the time, effort, and dangers of manually establishing and maintaining these power systems. With the use of light detection and ranging (LiDAR), inertial measurement units (IMU), global positioning systems (GPS), and camera vision, these mobile ground robots can search for a safe path to travel between the nodes, autonomously map their environment, and connect nodes in an electrical microgrid [5]. Using these onboard sensors, the UGV autonomously determines the shortest safe path and follows it. Once they reach an objective, the UGV deploys connectors to transmit power between the grid nodes. An example of two distributed UGVs and two loads in need of power are shown in Fig. 1. In an indoor setting with few obstacles, deployed cable can be spooled out passively as the UGV travels. In less forgiving outdoor environments, natural or man-made obstructions such as branches, trees, and buildings prevent this from being a realistic approach Other work has been performed which deploys cable with a mechanism which maintains a taut cable between control points in the environment [6], but for this application cable tension causes impassable segments in the operating field for other mobile agents. These impassable segments limit other mobile agents from connecting to other loads or require more time and energy to navigate around them to make these connections. Too much slack deploys an unnecessary amount of cable and causes potential for entanglement with the UGV. This problem is solved by controlling the deployment rate of the cabling as a function of the speed the UGV so the cable is laid on the ground without tension, or slack, along the track of the UGV. Retracting the cabling is accomplished by retracing the path the UGV followed, recoiling as a function of UGV speed of travel. By requiring the cable deployment speed to be adjustable across the range of expected UGV speeds, the UGV can maintain its existing planner for smooth motions through the operational environment, with the cable drive system achieving the slack-cable constraint. Cable management hardware which performs these tasks, the “Adjustable Cable Management Mechanism” (ACMM), enables these cabling operations that prevent the cable from being pulled out of line, snagging on nearby obstructions, or caught in the UGV as it is deployed. The utility of the mobile microgrid system hinges on accurate and efficient control of cable deployment/retraction.

Microgrids require reconfiguration to meet dynamic load conditions when nodes will need to be disconnected (shed) from the network, or when the grid needs to be reconfigured to accommodate different loads/sources. Once a load has been disconnected the retraction process can begin to recover deployed cable. Recoiling the cable autonomously allows for a single UGV to perform several connections in a different order or at a different location. Previous research has used tether systems as a means of guidance and used constant tension as a form of feedback in dimly lit environments where vision systems are not suitable, such as on the surface of Mars [7]. This however limits the terrain and complexity of the route. Our project focuses on retracing a cables path by following the steps used to create it and recoiling the cable along the way such that the cable is not allowed to tangle on obstacles. Other projects have utilized internal recoiling systems that are entirely self contained and use a series of gears to windup and release a tether from its storage reel. This solution, while effective for specific cable/ tethering needs, does not offer interchangeable tether options and depends on a complex and expensive set of gears to operate [7]. A similar design of a tether control system, used for lowering exploratory robots into Volcanoes, uses a feed through design with friction based attachment to the tether. This design was effective in controlling the release and retraction of the tether [8]. NASA’s Jet Propulsion Lab (JPL) has investigated a design for jumping robots that utilize recoiling tether with precision tracking of its movement [9]. An encoder is connected to the feed wheel to track the remaining and deployed tether. The ACMM follows a similar method of tracking by integrating the rotations counted by its stepper motor, allowing a precise measure of the deployed length of tether at all times, but contrasts from this work by imposing the speed control only on the tether rather than on the motion of the chassis. All the prior designs mentioned have a common similarity, they all appear to have been custom-designed to fit the user-specific application. It is likely that much time and resources could have been saved if complete, descriptive, open-source designs were available as a reference and inspiration. This is part of the goal in sharing the approach in this design.

Commercially available options for motorized spools exist. In majority, they are large industrial spools made for very high power applications and use brushed DC motors to quickly spool up and down cables. While these products are conducive to industrial operations, they are expensive in small quantity and too heavy for deployment on research-scale UGV’s. Our goal is to deploy 10-gauge electrical cabling from an unmanned ground vehicle, the Clearpath™ Husky. Limitations exist on the amount of cable available to connect the nodes. A cable capable of transferring 15 amps was selected for use with larger loads. We used a 75 foot cable spool to supply the UGV with this cabling. With such a limited amount of cable, it is important to know the exact amount remaining and to minimize usage. To determine the appropriate speed to move the cable, the turn radius of the vehicle and current directional speed are accounted for. The control on the ACMM inputs the velocity of the UGV and continually adjusts the cable extrusion motor speed.

## 2 Hardware description

**Fig. 2:**
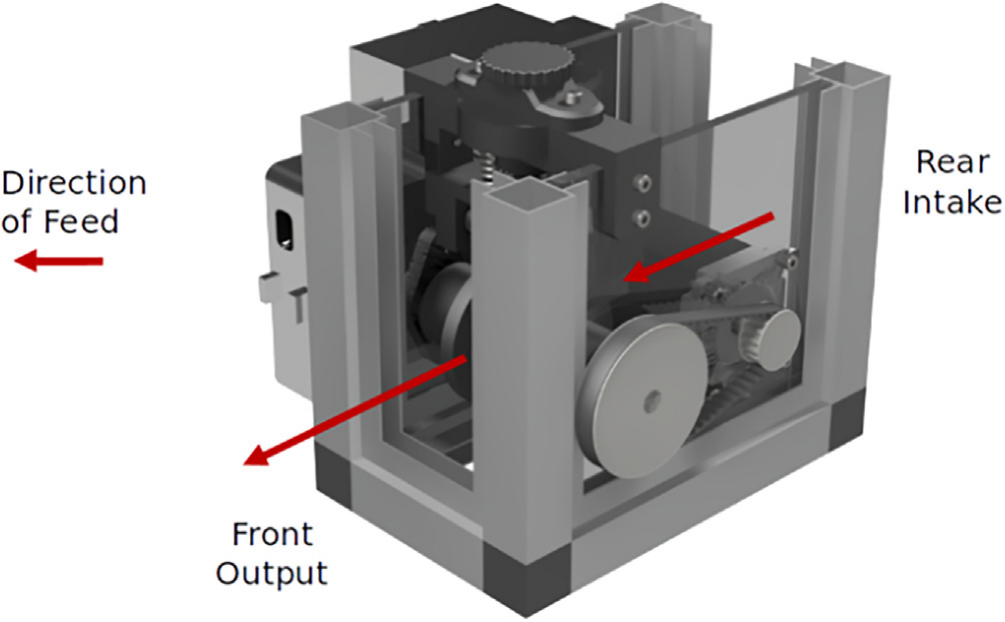
Complete assembly of the ACMM.

**Fig. 3:**
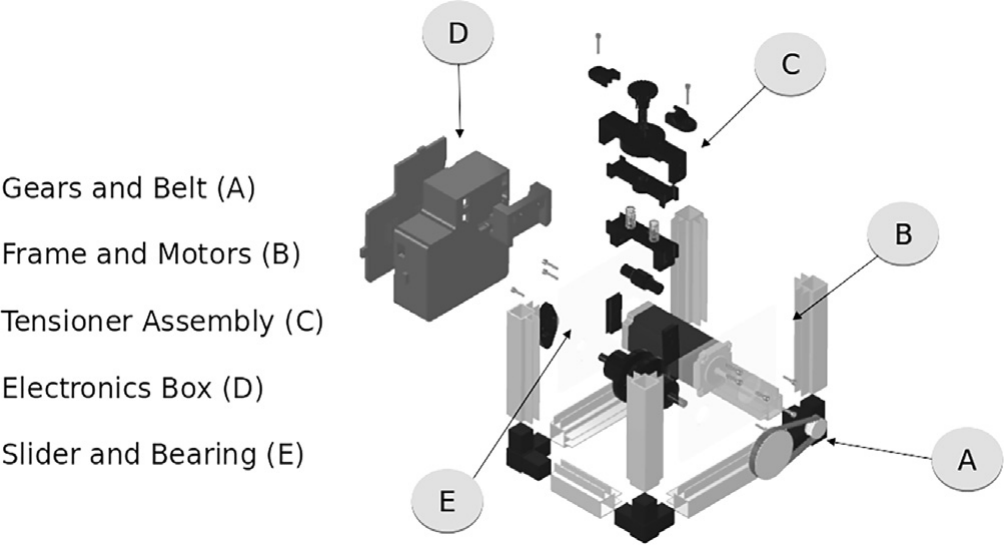
Full assembly explosion and subsections.

**Fig. 4:**
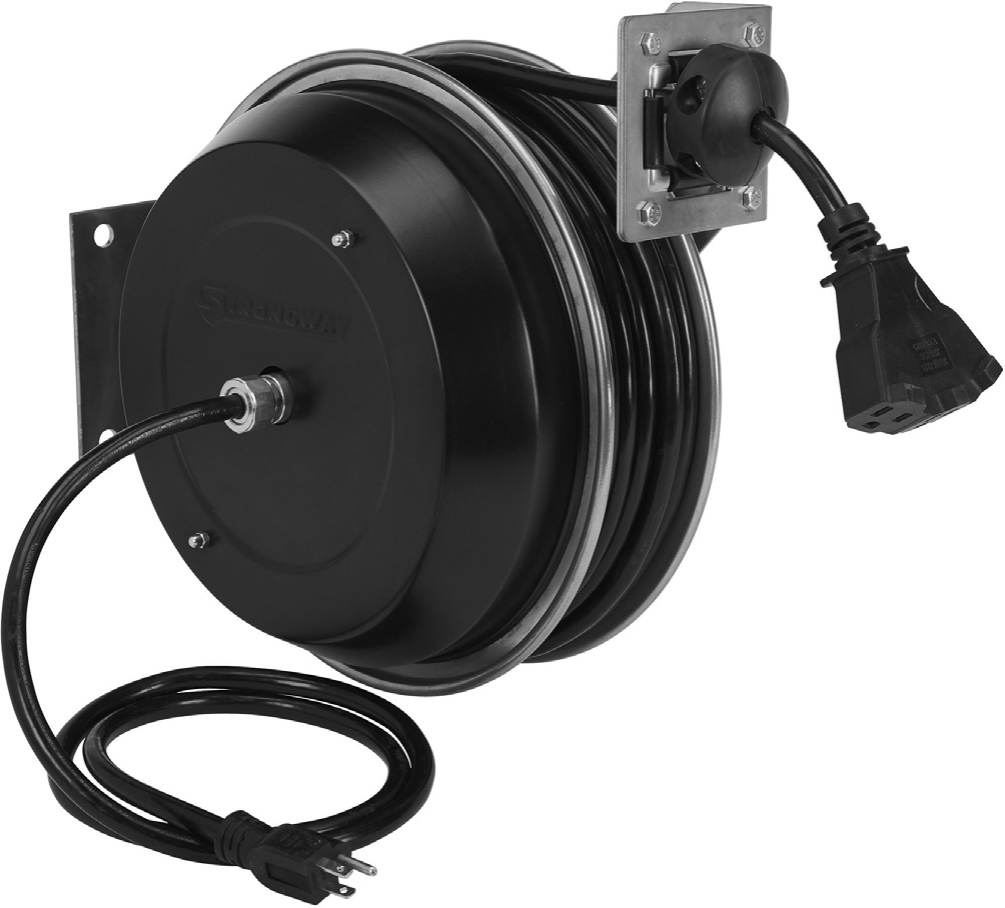
COTS cable spool.

Attempts to find an existing hardware solution suitable for the scale of a mobile microgrid UGV revealed a lack of COTS options. Powered cable control systems were too large, too heavy, and/or prohibitively expensive. Powered cable control systems could weigh over 200 lbs and cost USD1,600+. Creating a solution that provided precise control with a small footprint and mass was needed to fit on our project UGV’s payload bay, leaving room for the necessary sensor array and cable spool. The UGV has a weight limit of 150 lbs under optimal conditions; the ACMM cable extruder is a lightweight 5 lbs to keep the UGV unencumbered. The cable spool is the largest contribution to weight, with cables suitable for specifications weighing over 50 lbs for 70 ft length. Our solution requires an 8in×5in footprint in the payload bay, runs off a 24 V power supply, and can produce 15 lbs of retraction force on a variety of wire sizes. It consists of mostly 3D printed components and low-cost materials, making it accessible to many users. This lightweight platform can be mounted on any surface and can be used with many types of retracting spool systems. The type of cable will determine the type of spool needed; this system is intended to work with a spring-powered spool as described in the Bill of Materials. The following diagrams detail the components arrangement for assembly. The sections are broken into the four parts for better viewing. The complete assembly is shown in Fig. 2, with an exploded view of the assembly shown in Fig. 3. Fig. 4 shows the COTS cable spool which is integrated with the ACMM.

The cable spool in Fig. 4 was purchased from Northern Tool and has a spring-powered recoil which reaches 15 lbs at full extension of 75 ft. This spool helps maintain proper cable management, adding to the tension when recoiling and eliminating cord tangle. Other COTS variants of this product could be substituted dependent on local suppliers or availability.

Primary advantages and features of the ACMM are:

• Precise control to ±1in/70feet
• Speed adjusts to the deploying vehicle up to a top speed of 3.3 ft/s
• Small footprint, $<1\;{\text{ft}}^{3}$
• Applicable to cable gauges 8–15 awg
• Significant cost savings over comparable motorized cable reels
• Compatible with a range of cable reels and sizes
• Scalable and customizable to serve different applications
• Cost reduction of approximately USD1000 compared to similar powered cable spools

The ACMM works by applying a large force to the cable, trapping it between the drive roller attached to the motor and to a free roller which is pressed down by a one-way tightening bolt adjusted from the top. Tension can be adjusted until the cable does not lose traction. The friction on the cable is dependent on the tension between the spur roller and the free roller which clamp the cable. The spur roller is rotated with the geared-up motor by way of a keyed 10mm drive rod. To adjust the tension, a bolt is turned to increase the clamping pressure. Because a stepper motor is used, a holding torque exists while activated, preventing the cable from being deployed inadvertently. Proper adjustment ensures no slip occurs between the cable and spur roller, enabling precise accuracy in feed velocity. It is therefore very important to manually adjust the tension during commissioning so that slipping does not occur.

### 2.1 Electronics

The electronics system for the ACMM is composed of 4 parts:

• NEMA 24 stepper motor
• DMT542T stepper driver
• Arduino Uno
• Rotary Encoder

**Fig. 5:**
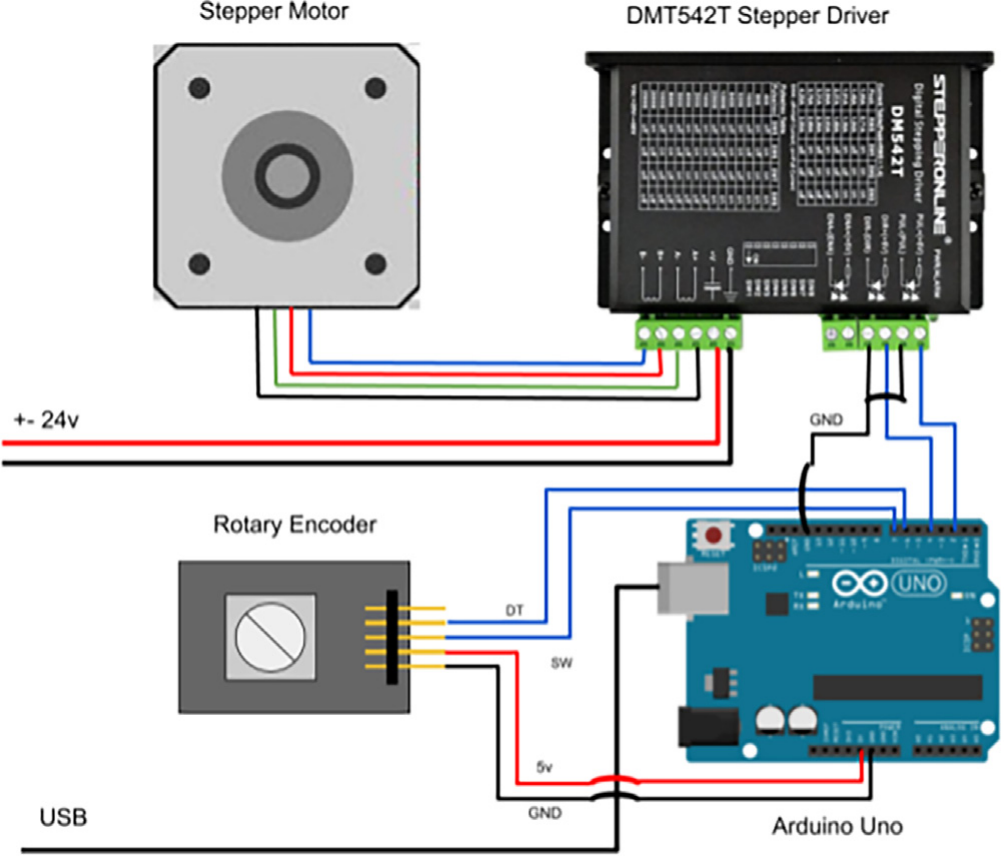
Wiring Diagram.

The Nema 24 stepper motor runs on $24V_{\mathrm{dc}}$. The associated motor controller must be tuned to the appropriate PWM settings. The controlling software is written using the Arduino language. This motor can provide a 15 lb holding force to the cable. Control software transforms the linear and angular velocity of the UGV to a linear velocity request for cable extrusion at the stepper motor. Because step rate is deterministic up to the rated slew speed of the stepper motor, the extrusion rate is known with very high accuracy and open loop control for the motor velocity command is sufficient. The system is connected to an Arduino Uno microcontroller. This microcontroller was chosen for the well-established open-source software support for the hardware drivers used in this design. Anyone considering adapting this design to their needs should evaluate which microcontroller will provide sufficient computational power and peripheral capacity for all of their hardware subsystems, as well as the constant advancement of capability at a given price-point. The Arduino is wired into the motor controller with input from an encoder for manual speed adjustment, and a mode selection switch, Fig. 5. The electronics are housed opposite the gears in the printed electronics enclosure.

### 2.2 Control code

**Fig. 6:**
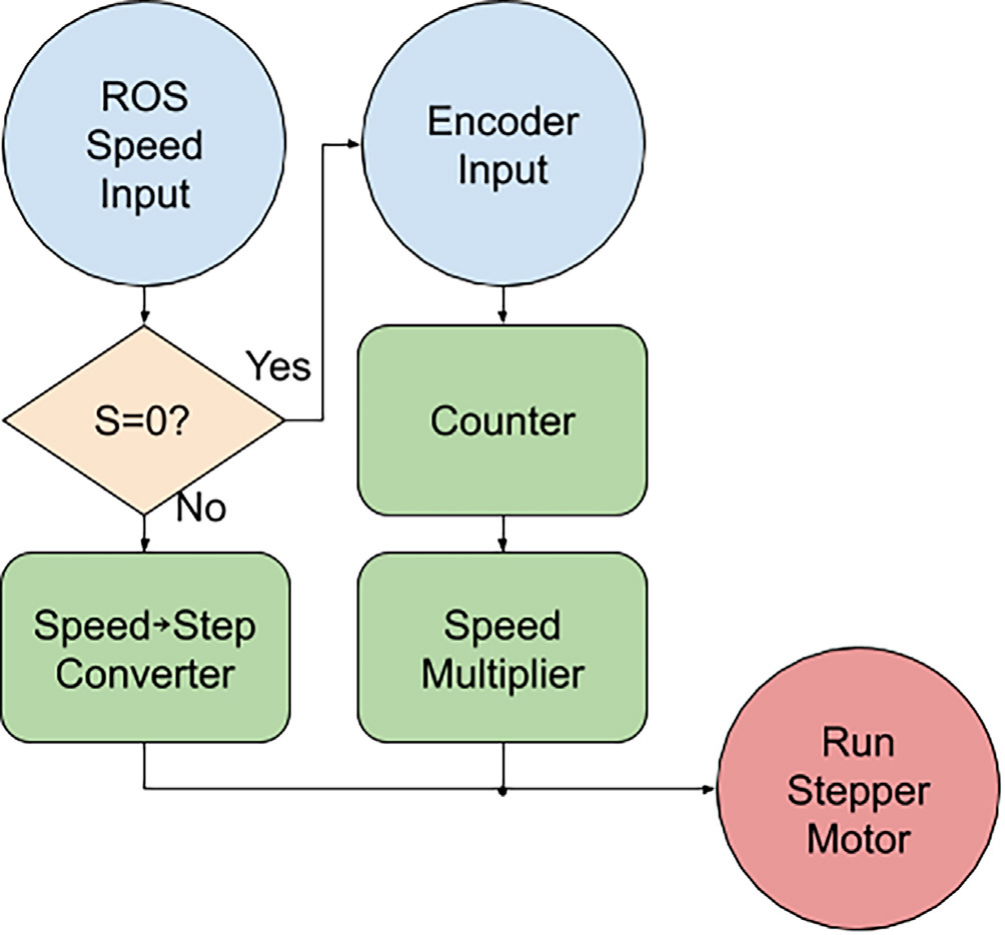
Function Block diagram of ACMM control code.

Provided in the repository is control code for the ACMM, as an Arduino.ino file. The code accepts speed set-point inputs from both ROS and an encoder. The speed set-point is converted into the steps-per second rate required by the stepper motor controller. If there is no ROS input, the ACMM can be controlled manually through an encoder. The operating principle behind the encoder code is the use of the difference in state generated by the rotary encoder to increment a counter higher or lower. This counter sets the speed of the stepper motor via a preset speed multiplier and the counter’s magnitude. It is important to note that the acceleration of the stepper motor and its maximum speed fixed parameters in the code which cannot be adjusted in real-time. Fig. 6 is a function block diagram for the control code.

## 3 Design files

To reduce the costs, weight, and complexity of construction, many of the components are 3D printed out of PLA filament using a Cetus MK3 3D Printer. The.STL files and drawings for reference are available as supplemental material with this article. See the 3D Printed Components BOM. The components that were not 3D printed have been selected commercially and purchased in majority through McMaster Carr.

**Table:** 

**Design filename**	**File type**	**Open source license**	**Location of the file**

Bearing Block	STL	CERN OHL	10.17605/OSF.IO/8WKJT
Box Lid	STL	CERN OHL	10.17605/OSF.IO/8WKJT
Box Mount	STL	CERN OHL	10.17605/OSF.IO/8WKJT
Electronic Box	STL	CERN OHL	10.17605/OSF.IO/8WKJT
plate 1	DXF	CERN OHL	10.17605/OSF.IO/8WKJT
plate 2	DXF	CERN OHL	10.17605/OSF.IO/8WKJT
Roller Housing	STL	CERN OHL	10.17605/OSF.IO/8WKJT
Roller	STL	CERN OHL	10.17605/OSF.IO/8WKJT
Screw Mount	STL	CERN OHL	10.17605/OSF.IO/8WKJT
Screw Retainer	STL	CERN OHL	10.17605/OSF.IO/8WKJT
Screw	STL	CERN OHL	10.17605/OSF.IO/8WKJT
Slider Rail	STL	CERN OHL	10.17605/OSF.IO/8WKJT
Spring Carrage	STL	CERN OHL	10.17605/OSF.IO/8WKJT
Spur Roller	STL	CERN OHL	10.17605/OSF.IO/8WKJT
Speed_Control	INO	GNU GPL	10.17605/OSF.IO/8WKJT
extruderWiring	PDF	CERN OHL	10.17605/OSF.IO/8WKJT

The *Box Lid*, *Box Mount*, and *Electronics Box* comprise the enclosure for the stepper driver, Arduino, and associated wiring and components. Arduino software is provided in *Speed*_*Control*, with *extruderWiring* diagram for completing the electrical system. CNC cut panels, *plate 1* and *plate 2*, align the mechanical components. The friction adjustment on the drive pulley consists of 3D printed components: *Screw Mount, Screw Retainer, Screw, Slider Rail, Spring Carrage* and *Spur Roller*. The drive pulley includes *Roller Housing* and *Roller*, with the stepper motor supported by the *Bearing Block*.

## 4 Bill of materials

**Table:** 

**#**	**Component**	**Qty**	**Unit Cost (USD)**	**Total Cost (USD)**	**Material Source**	**Material Type**

1	Stepper Motor	1	39.99	39.99	StepperOnline	N/A
2	Motor Controller	1	39.89	39.89	StepperOnline	N/A
3	10 mm shaft bearing	1	6.89	6.89	McMaster	Steel
4	$2.5^{\prime \prime }$ Diam. Pulley	1	21.00	21.00	McMaster	Al
5	$1.5^{\prime \prime }$ Diam. Pulley	1	9.89	9.89	McMaster	Al
6	$12^{\prime \prime }$ Drive Belt	1	5.87	5.87	McMaster	Rubber
7	10 mm × $1^{\prime }$ Rod	1	2.01	2.01	McMaster	Al
8	$0.25^{\prime \prime }\times 1^{\prime }$ Rod	1	1.81	1.81	McMaster	Al
9	$3^{\prime \prime }$ 8 lb/in Spring	2	varies		Hardware	Steel
10	$12^{\prime \prime }\times 12^{\prime \prime }\times 1/4^{\prime \prime }$ Sheet	1	15.89	15.89	McMaster	Polycarb.
11	$4^{\prime }$ Double Corner Slotted Rail	1	15.97	15.97	McMaster	Al
12	3way 90° Elbow	4	6.50	26.00	Mcmaster	Plastic
13	M4 × 18 mm Q. 100 Socket Cap Screw	1	13.00	13.00	McMaster	Steel
14	$80^{\prime }$ Cable Reel	1	299.99	299.99	Northern Tool	Multiple

## 5 Build instructions

To construct the ACMM, begin by milling out the side panels on to polycarbonate sheets or similar $1/4^{\prime \prime }$ material. Print the parts listed in the 3D printed components BOM and use high fill settings.

**Fig. 7:**
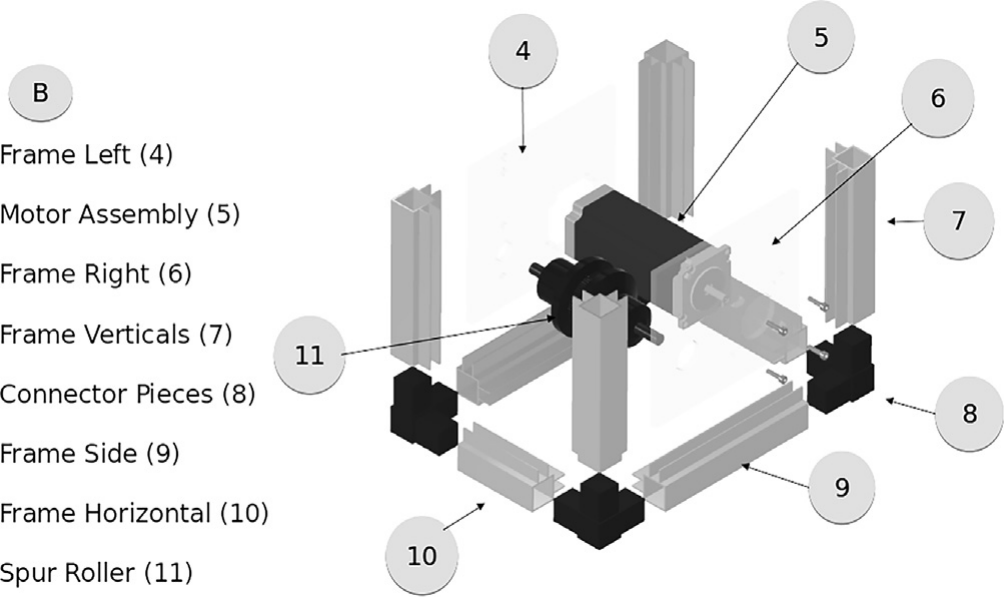
Frame and Motor assembly.

The frame, shown in Fig. 7, is composed of a slotted aluminum extrusion. To assemble these pieces cut pieces into three different lengths. Cut the extrusion pieces to the following lengths:

• $4^{\prime \prime }$ Frame Verticals – 4 parts
• $3^{\prime \prime }$ Frame Horizontals – 2 parts
• $4^{\prime \prime }$ Frame Sides – 2 parts

Cut $0.5^{\prime \prime }$ of the slotting at each of the side and horizontal extrusion pieces so they fit together at the corners. Hammer the extrusions into the corner pieces.

**Fig. 8:**
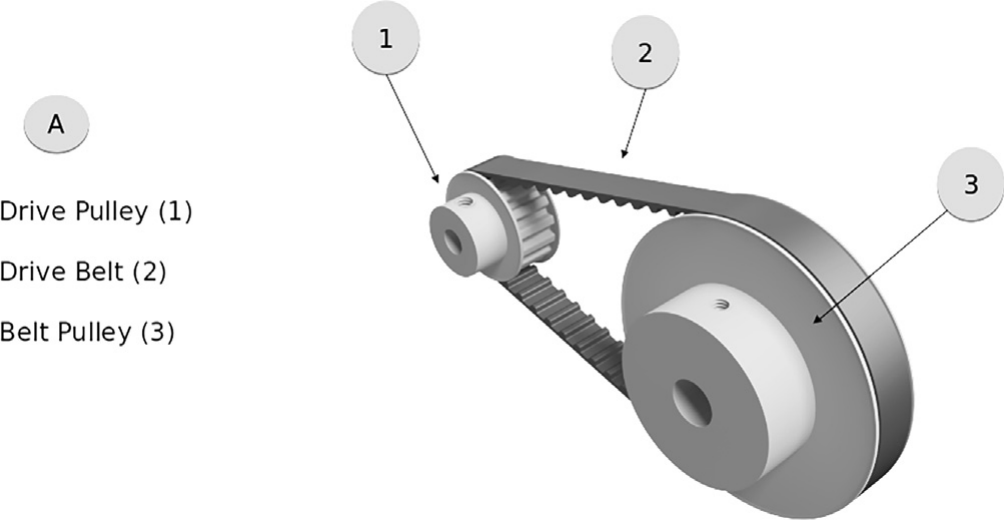
Pully and belt drive system.

Add a key cut to the 10 mm rod (x3). The pulley system, shown in Fig. 8, provides geared down power from the motor to the drive. The centers of both pulleys will need to be bored out to 10 mm before they will fit to the selected rod and motor. Connect the Larger pulley onto the 10 mm rod and fasten the screws (x4). Slide the keyed rod into the center roller (x5). Place the cut polycarbonate sheets into the slots of the frame (x6) Slide the motor into place, add loose bolts, then fasten the smaller pulley onto it (x7).

**Fig. 9:**
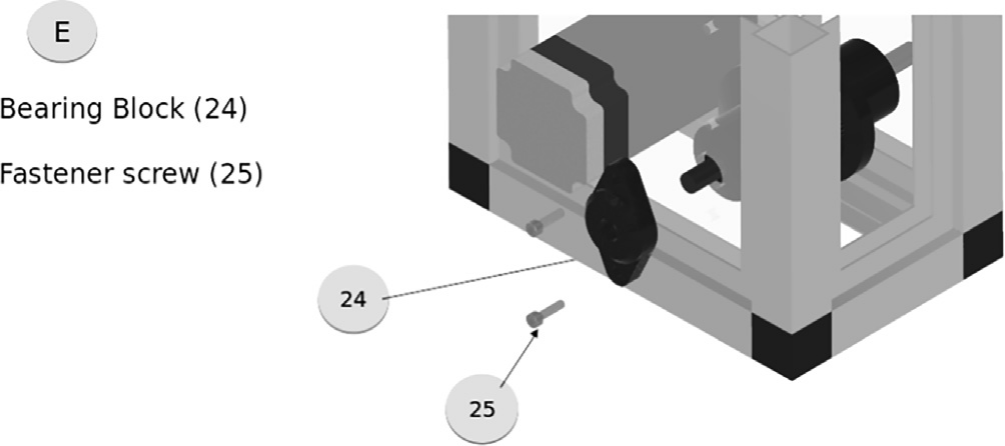
Bearing Block Assembly.

The bearing block, shown in Fig. 9, holds the bearing that engages with the 10 mm rod running through the roller. This is fastened to the frame by two screws. Place rollers into (a), lubricate rails with lithium grease (x8). Install and fasten top support and sliders (x9).

**Fig. 10:**
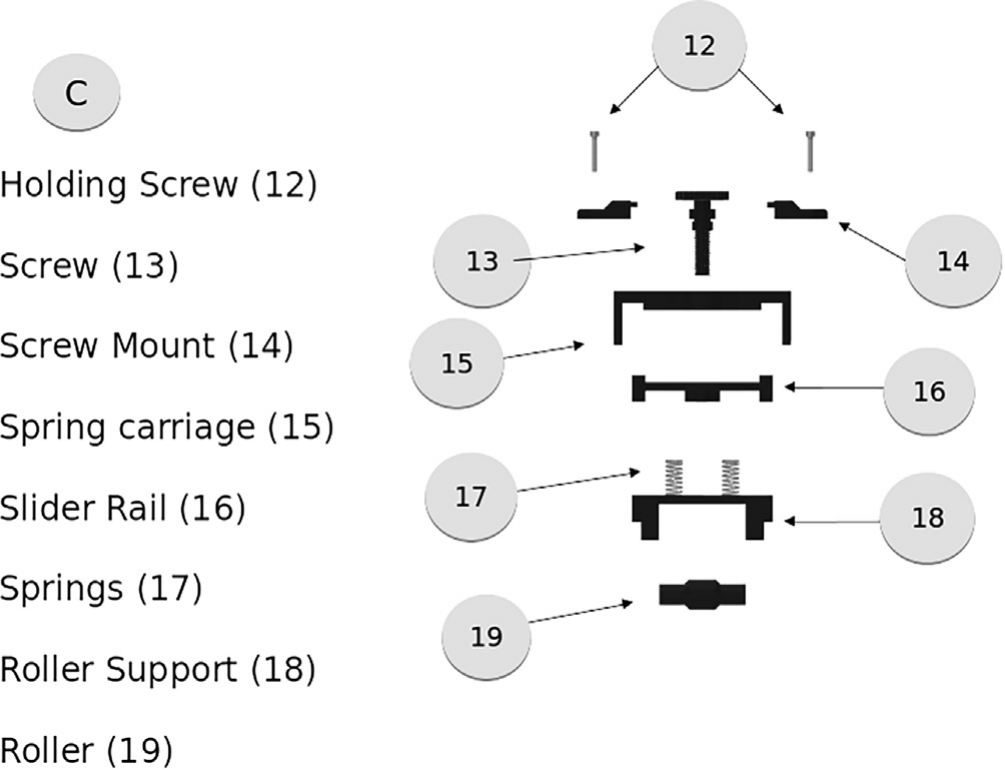
Tensioner Assembly.

The tensioner assembly, shown in Fig. 10, is composed of 3D printed components that apply pressure on the cable held between the roller and the spur roller which is driven by the motor. The tensioner is adjusted by the screw, which tightens with a counterclockwise twist. The slider rail should be lubricated with graphite powder or lithium grease. A short section of $0.25^{\prime \prime }$ rod is used between the roller support and the roller allowing it free spin. These components should be printed with high infill to ensure maximum strength.

**Fig. 11:**
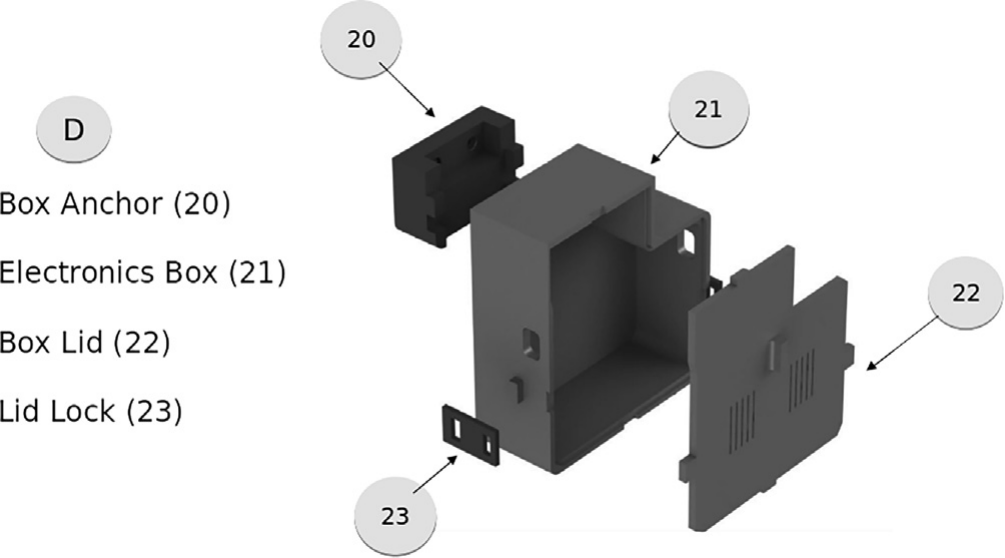
Electronics Enclosure.

The electronics box, shown in Fig. 11, is designed to hold the needed components for the motor controller and arduino board. There is additional space for any added components and spacing for wire routing. The box anchor must be adhered to the Electronics box after the Box anchor has been placed. This is best done with a 5 min epoxy.

Complete the wiring according to the diagram in Fig. 5. To prepare the cable spool for deployment, remove the locking mechanism located opposite the power input. This will prevent the spool from locking when the retraction process begins. Change out the power outlet end as needed for your project. Mounting of this component will be vehicle dependent, but the output path of the spool must align with the input of the ACMM.

## 6 Operation instructions

While it is outside the scope of this article to define all the possible integration steps of this ACMM system with a host UGV, a brief handling of some contingencies is merited. This system is deployed in practice with a visual feedback controller for the cable trajectory as it exits the UGV. This controller runs in a Robot Operating System (ROS) high-level state machine, and can trigger a failure condition if it identifies that the cable deployment system is not maintaining its nominal state, i.e. the cable is not paying out or retracting quickly enough to match the speed of the UGV chassis. This type of fault could occur from an unlikely entanglement or binding of the cable in the storage reel, or from a failure of the UGV navigation controller to sufficiently avoid obstacles, resulting in entanglement of the cable. In any of these conditions, the vehicle must be stopped, as well as the ACMM.

In regard to the fundamental operation of the ACMM subsystem, the following steps should be addressed:

• Securely mount the frame to the UGV.
• Securely mount base of reel behind the feed in location, leaving 3 inches of space for clearance.
• Feed the end of the desired cable to extrude from its reel through the back side of the extruder roll and tighten the tensioner to apply the desired amount of force. This tension required will depend on the cable size and reel used.
• Connect the extruder motor control to power and turn the encoder clockwise slowly to begin extruding the cable from the intake location (back) to the output side (front)

Safety Concerns:

• Keep loose hanging clothing and hair away from the extruder.
• Do not leave on when not in use to prevent motor overheating.
• Ensure the device is firmly attached before using.
• Keep hands away from the device while running.

## 7 Validation and characterization

**Fig. 12:**
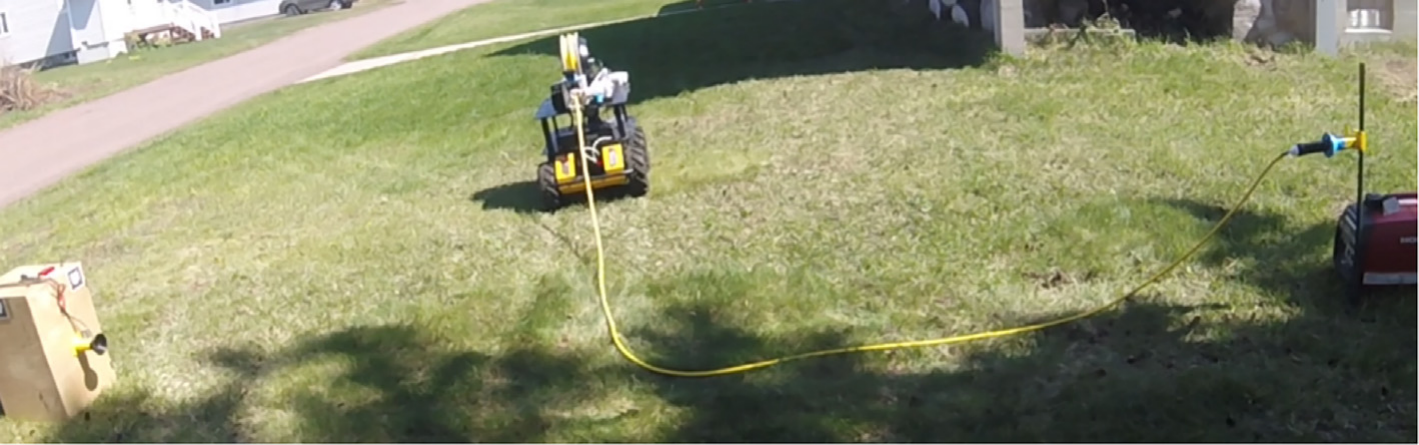
A demonstration video of the ACMM on a UGV includes, from left to right, an infrastructure load, the cable following the track of the UGV through the environment, and a genset.

Verification of the design is provided by a physical operation demonstration. Video of the demonstration is included with the supplemental material to this article, available athttps://doi.org/10.17605/OSF.IO/8WKJT. This demonstration was performed in an outdoor environment. The UGV departs a power source location, and traverses through the region to rendezvous with an infrastructure load, Fig. 12. (The video is presented at 4x speed for brevity.) While the navigation, docking, and power connector coupling controllers are outside of the scope of this manuscript, some comments about the cable deployment are merited. The control system uses a feedforward-feedback controller, with the UGV velocity command as an input to the feedforward component of the control signal, and a visual cable angle estimate at the exit from the ACMM wrapped in a PID feedback loop. In the video, the feedback controller dominates when the vehicle is stationary, and then works with the feedforward control to provide disturbance rejection as the vehicle traverses, such as thick grass or small hills affecting the vehicle performance. Four types of maneuvers are captured in this demonstration video: forward progression while deploying cable, stopping the UGV and cable, retracting the cable while the UGV reverses back to its starting point, and a right turn while deploying cable on the ground in the track of the UGV.

This design mixed standard off-the-shelf components with some 3D printed mechanisms, representing a trade-off between durability vs cost and ease of fabrication. The result is a design which is suitable for use in the robotic controls development iterations, with demonstrations and validation/robustness testing. For long-term deployment, one limitation of the design is that some of the 3D printed components in the tensioning assembly may need to be replaced by machined alloy components.

## Declaration of Competing Interest

The authors declare that they have no known competing financial interests or personal relationships that could have appeared to influence the work reported in this paper.
